# Comparative analysis of whole exome sequencing kits for the canine genome

**DOI:** 10.1371/journal.pone.0312203

**Published:** 2024-11-04

**Authors:** Jinhee Jang, Yong-Jik Lee, Soohyun Ko, A. M. Abd El-Aty, Ibrahim Gecili, Ji Hoon Jeong, ChangHyuk Kwon, Tae Woo Jung

**Affiliations:** 1 GenesisEgo, Seoul, Republic of Korea; 2 Department of Pharmacology, College of Medicine, Chung-Ang University, Seoul, Republic of Korea; 3 Department of Pharmacology, Faculty of Veterinary Medicine, Cairo University, Giza, Egypt; 4 Department of Medical Pharmacology, Medical Faculty, Ataturk University, Erzurum, Türkiye; 5 Department of Global Innovative Drugs, Graduate School of Chung-Ang University, Seoul, Republic of Korea; University of Veterinary Medicine Vienna: Veterinarmedizinische Universitat Wien, AUSTRIA

## Abstract

**Objectives:**

As the public’s interest in companion dogs grows, health issues in these animals are also emerging, necessitating the optimization of whole exome sequencing (WES) as a valuable method for disease prediction. While WES targeting the human genome is well established, WES targeting the canine genome is understudied, and there is a need to find effective analysis kits.

**Methods:**

We compared and analyzed the performance of three WES kits from Twist and Agilent using the canine genome as the target to perform genetic analysis of canine diseases effectively. The levels of total reads, the duplication rate, and variant calling in canine genomic DNA samples from seven healthy dogs (three beagles, one bichon fry, one maltese, one welsh corgi, and one mixed breed) without any interventions were examined through WES via Twist and Agilent kits.

**Results:**

We found that while Twist had the lowest total read number, the number of reads in the SSXT series was significantly (*P*<0.05) greater. Twist showed low evenness and high standard deviation, but the SSXT series showed relatively high evenness. Compared with Twist, the SSXT series from a depth of 30× presented a significantly (*P*<0.05) greater target ratio. Among the four kits, the significantly lowest duplicate ratio was confirmed for SSXT (O/N) (30% lower than Twist).

**Conclusion:**

The most important performance of the kit, the number of variants detected, was 48,302 for Twist and 130,506 for SSXT (O/N). On the basis of the performance comparison results, SSXT (O/N) was found to have the best performance.

## Introduction

With the increasing popularity of companion dogs as family members, there is increasing interest in predicting and managing genetic diseases through genome analysis [1, 2]. Hence, there is a need for a cost-effective and accurate genome analysis method for companion dogs, considering the growing social trend of improving their health and quality of life.

Massively parallel sequencing has ushered in the era of comprehensive genomics by significantly reducing costs and time requirements. Despite rapid technological advancements, whole-exome sequencing (WES) methods are becoming increasingly efficient [3]. WES has become a common choice for genetic testing because of its focus on the protein-coding regions (exons) of genes in the genome, which make up only 1–2% of the human genome but contain up to 85% known variants with diagnostic significance [4]. Notably, WES is also more cost-effective, being 3–5 times less expensive than whole-genome sequencing is [5]. Furthermore, WES has demonstrated its efficiency as a diagnostic tool for multiple traits, making it particularly valuable in the field of human clinical genetics.

Multiple commercially available kits are used for WES, which employ similar protocols for target enrichment. These protocols typically involve hybridization between exon sequences and biotin-conjugated DNA or RNA probes, followed by capture via streptavidin-coated magnetic beads. A recent study by Belova et al. revealed that the quality of data obtained from WES can vary depending on the type of probes used in the human genome [6].

Our study aimed to identify the most optimized kit method for WES of the canine genome. We performed a comparative analysis of three whole-exome capture platforms using SSXT as a reference because of its known high data quality and accuracy.

## Materials and methods

### Sample collection

#### Ethics approval for laboratory animal studies

Dog studies were conducted with approval from the Institutional Animal Care and Use Committee of HLB BioStep Co., Ltd. (Incheon, Republic of Korea) (Approval No. IACUC# 22-KE-0348). All samples used in this study were purchased from HLB BioStep Co., Ltd.

#### Blood sample collection

We obtained three blood samples from three healthy experimental beagles via CE-IVD Cell-Free DNA Collection Tubes (Cat. No. 0778566600, Roche Diagnostics, Laval, QC, Canada). The plasma was separated from the blood samples within 4 h of collection to isolate buffy coats, which were then stored at -70°C until DNA extraction.

#### Buccal swab sample collection

Six buccal swab samples were collected via an Accubuccal collection kit (Cat. No. ACN 21.01; Accugene, Incheon, Republic of Korea), with the consent of each owner. The oral samples were stored at room temperature until DNA extraction. Information on the sample types is summarized in Table 1. Information on the experimental sets is organized in S1 Table.

**Table 1:** Information on sample types.

sequencing name	breed	Origin	sample name
B1	Beagle	Blood	Beagle #1
B2	Beagle	Blood	Beagle #2
S1	Beagle	Buccal Swab	Beagle #1
S2	Beagle	Buccal Swab	Beagle #2
S3	Bichon Frise	Buccal Swab	Bichon Frise #3
S4	Mix	Buccal Swab	Mix #4
S5	Maltese	Buccal Swab	Maltese #5
S6	Welsh Corgi	Buccal Swab	Welsh Corgi #6
B7	Beagle	Blood	Beagle #7

#### Genomic DNA isolation and preparation for WES

The DNA from the blood samples was isolated via the QIAamp DNA Blood Mini Kit (Cat. No. 51104, QIAGEN, Hilden, Germany) [https://www.qiagen.com/us/products/discovery-and-translational-research/dna-rna-purification/dna-purification/genomic-dna/qiaamp-dna-blood-kits] following the manufacturer’s instructions. The DNA from the buccal swab samples was extracted via the Accubuccal DNA Preparation Kit (Cat. No. ACN 08.50, Accugene) [https://accugenelab.com/en/products/accuprep/accubuccal-dna-preparation-kit/]. The extracted genomic DNA was then fragmented to a size of 180–210 bp via a Q800R3 ultrasonicator (Qsonica, Newtown, CT, USA) to construct the library. The length of the fragmented DNA was confirmed via Agilent D1000 ScreenTape (Agilent Technologies, Santa Clara, CA, USA).

#### Library preparation, probe hybridization and sequencing

**Fig 1:**
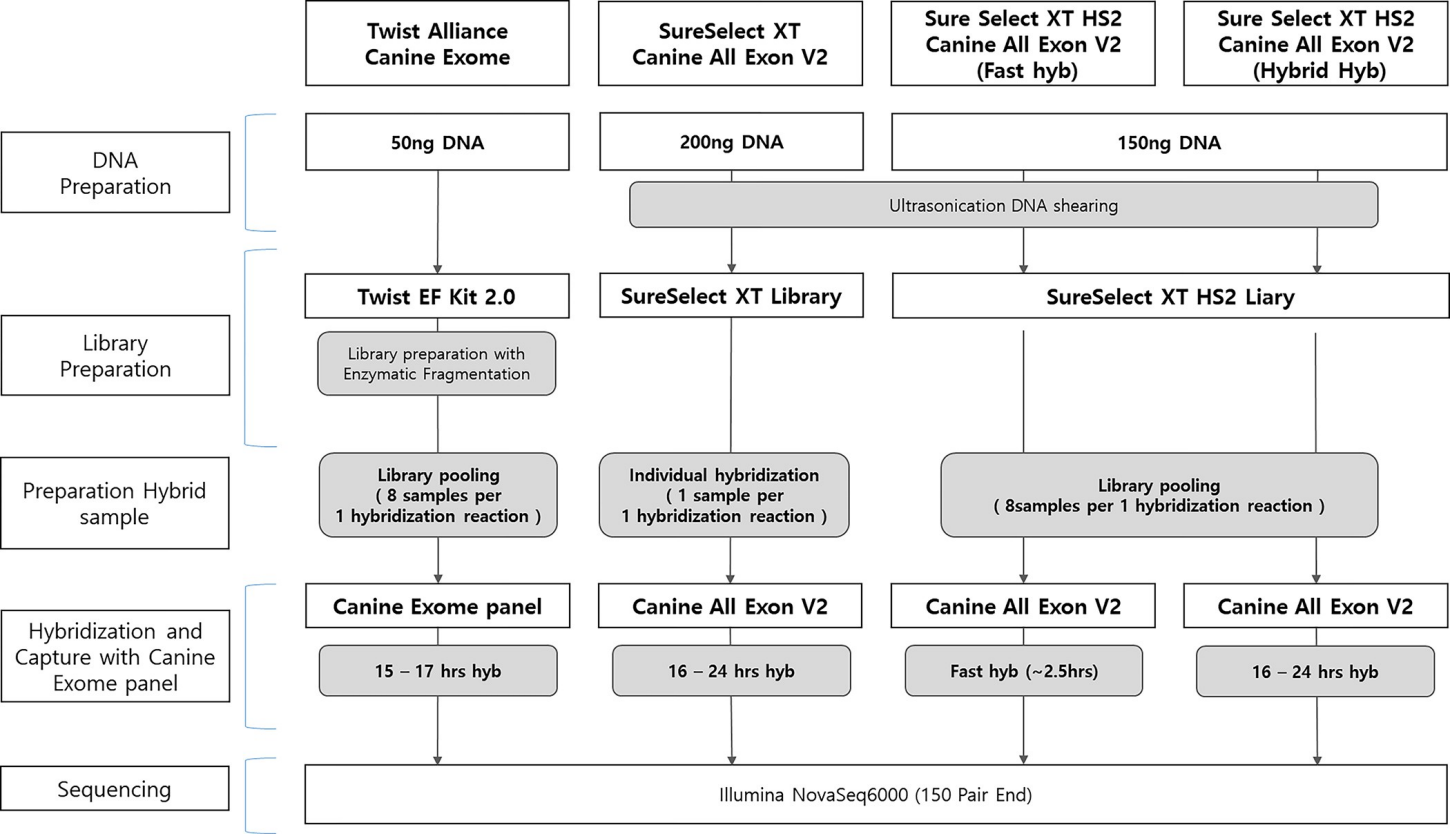
The design and workflow of the current study. Twist: Twist Alliance; SSXT: SureSelect XT; SSXT (Fast): SureSelect XT HS2 (Fast hyb); SSXT (O/N): SureSelect XT HS2 (Overnight hyb).

The construction of exome libraries was carried out via the Twist Alliance Canine Exome (Twist Bioscience, San Francisco, CA, USA) [https://www.biorxiv.org/content/10.1101/2024.05.19.594885v1] and SureSelect Community Design Canine All Exon V2 (Agilent) kits [7–9], following the manufacturer’s instructions. The resulting exome libraries were then sequenced via the Illumina NovaSeq 6000 system. The overall procedure for library construction and probe hybridization is shown in Fig 1. The protocols of each kit are as follows: Twist (kit info: https://www.twistbioscience.com/products/ngs/alliance-panels#tab-4; Library: https://www.twistbioscience.com/resources/protocol/library-preparation-ef-20-enzymatic-fragmentation-and-twist-universal-adapter; Capture: https://www.twistbioscience.com/resources/protocol/twist-target-enrichment-standard-hybridization-v1-protocol). SSXT (Library and capture: https://www.agilent.com/cs/library/usermanuals/public/G7530-90000.pdf). SSXT Fast (Library and capture: https://www.agilent.com/cs/library/usermanuals/public/G9985-90000.pdf). SSXT O/N (Library and capture: https://www.agilent.com/cs/library/usermanuals/public/G9957-90000.pdf).

#### Analysis method for exome sequencing data

The sequences obtained were aligned to the CanFam3.1 reference genome via BWA-MEM [10], and the aligned sequences were saved in a technology-independent SAM/BAM file format [11, 12]. Duplicate fragments were marked and removed via Picard (version 1.9) (http://picard.sourceforge.net). After the mapping quality was evaluated and low-quality mapped reads were filtered out, paired-read information was examined to ensure consistency between mate-pair reads. Further processing included the removal of PCR duplicates, indel realignment, mate information fixation, base quality score recalibration, and variant quality score recalibration on putative SNVs and indels. The Strelka2 variant caller was used with default parameters to identify variants [13]. Variant filtering was applied uniformly across all the result files, with criteria such as "PASS" in the quality filter, a quality score of 10, and at least four supporting reads for alternative alleles. Annotation was performed via ANNOVAR and custom R scripts developed in-house [14].

### Statistical analyses

All the statistical analyses were performed via GraphPad Prism 7.0 software (GraphPad Software, San Diego, CA, USA). The results are presented as the means ± standard deviations (SDs). Each experiment was conducted eight times for statistical validity (except for SSXT). Statistical significance was determined via one- or two-way repeated ANOVA followed by Tukey post hoc tests.

## Results

### Feature comparison of the four whole-exome capture platforms

The study design and workflow are depicted in Fig 1 and S1 Fig, while Table 2 presents a comparison of the features of the four whole-exome capture platforms, including Twist and Agilent. Notably, on the basis of the SSXT platform, SSXT (Fast) and SSXT (O/N) have been developed by Agilent to reduce the amount of DNA required for library preparation and minimize experimental time and effort. While the bait length in all kits is 120 bp, Twist has a total bait length of 40.5 Mb, whereas Agilent kits have 43 Mb. Additionally, Agilent kits require ultrasonication for DNA fragmentation, whereas Twist employs an enzymatic reaction method. Twist also has a lower DNA input requirement of only 50 ng for library preparation, whereas Agilent kits typically require 150–200 ng. Furthermore, the probe hybridization incubation time for most kits ranges from 15 to 24 h, but the Agilent fast (SSXT) kit requires only 2.5 h, providing a notable time-saving advantage.

**Table 2:** The features of four kits for WES.

	Twist Alliance Canine Exome	Agilent		
		SureSelect XT Canine All Exon V2	Sure Select XT HS2 Canine All Exon V2	Sure Select XT HS2 Canine All Exon V2
			Fast hyb	overnight hyb
Bait type	dsDNA	ssRNA	ssRNA	ssRNA
Bait length (bp)	120	120	120	120
Total bait length (Mb)	40.5	43	43	43
Total target length (MB)	NP	NP	NP	NP
Method of library preparation	Twist EF Kit 2.0	SureSelect XT Lib kit	SureSelect XT HS2 Lib kit	SureSelect XT HS2 Lib kit
Fragmentation method	Enzymatic Fragmentation	Ultrasonication	Ultrasonication	Ultrasonication
DNA input for library preparation (ng)	50	200	150	150
Hyb incubation (hrs)	15–17	16–24	~2.5	16–24

### Target coverage efficiency of the four whole-exome capture platforms

**Fig 2:**
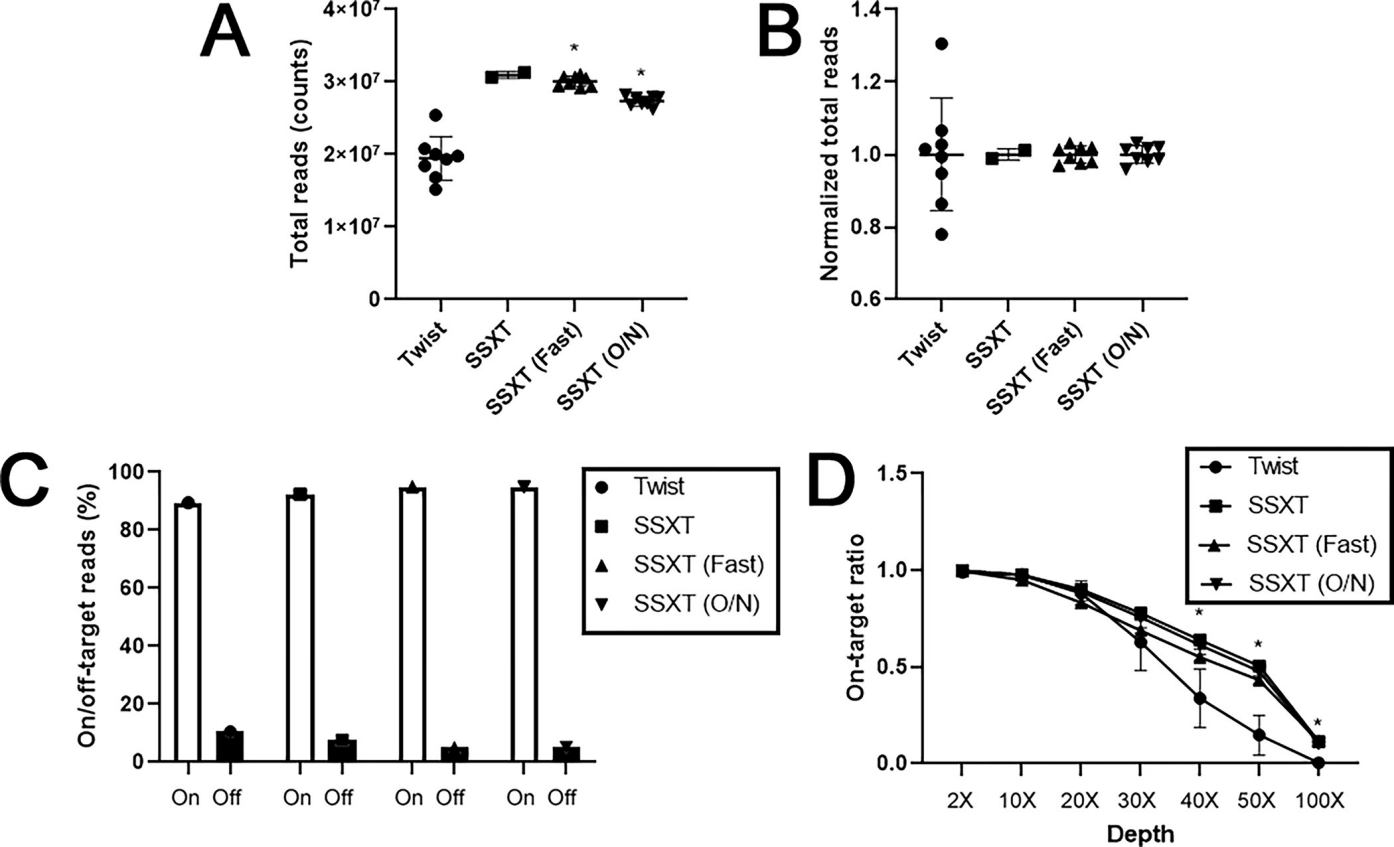
Target coverage efficiency of the four whole-exome capture platforms. (A and B) Total read counting analysis via Twist (n = 8), SSXT (n = 2), SSXT (Fast) (n = 8), and SSXT (O/N) (n = 8) kits. (C) On/off-target read analysis via Twist, SSXT, SSXT (Fast), and SSXT (O/N) kits. (D) On-target ratio analysis via Twist, SSXT, SSXT (Fast), and SSXT (O/N) kits. The means ± SDs were calculated from eight independent experiments (except for SSXT). *P < 0.05 compared with Twist. n = 8: six buccal swabs and two blood samples.

The level of reads, which represents the coverage of the target regions, was examined. Among the Agilent kits, SSXT presented the highest read level, whereas SSXT (O/N) presented the lowest (Fig 2A and S2 Table) (*P*<0.0001, R^2^: 0.8839). However, compared with those in Twist, significantly greater total read numbers were detected in SSXT (O/N). The evenness score, which is used to quantify the homogeneity of target coverage with sequencing reads [15], was analyzed via normalized read counts. Twist’s kit showed the largest deviation, whereas the Agilent kits presented relatively minimal variation, with the SSXT (O/N) kit showing the least variation and excellent evenness (Fig 2B). On-target reads refer to the mapped nonoverlapping reads that overlap with at least one base of the primary target region [16]. Twist’s kit had the lowest on-target read level at 89.46%, whereas SSXT (Fast) and SSXT (O/N) presented excellent values of 94.97% and 94.86%, respectively. Conversely, Twist’s kit had the highest off-target read level at 10.54%, whereas SSXT (Fast) and SSXT (O/N) presented low values of 5.03% and 5.14%, respectively (Fig 2C and S3 Table). The Twist kit showed excellent on-target ratio scores up to 20× depth, but a significant decrease (*P*<0.0001) was observed above 30× depth (Fig 2D). A comparison of six buccal swabs and two blood samples from each breed revealed no obvious differences (S5 Table).

### Duplicate ratios of the four whole-exome capture platforms

**Fig 3:**
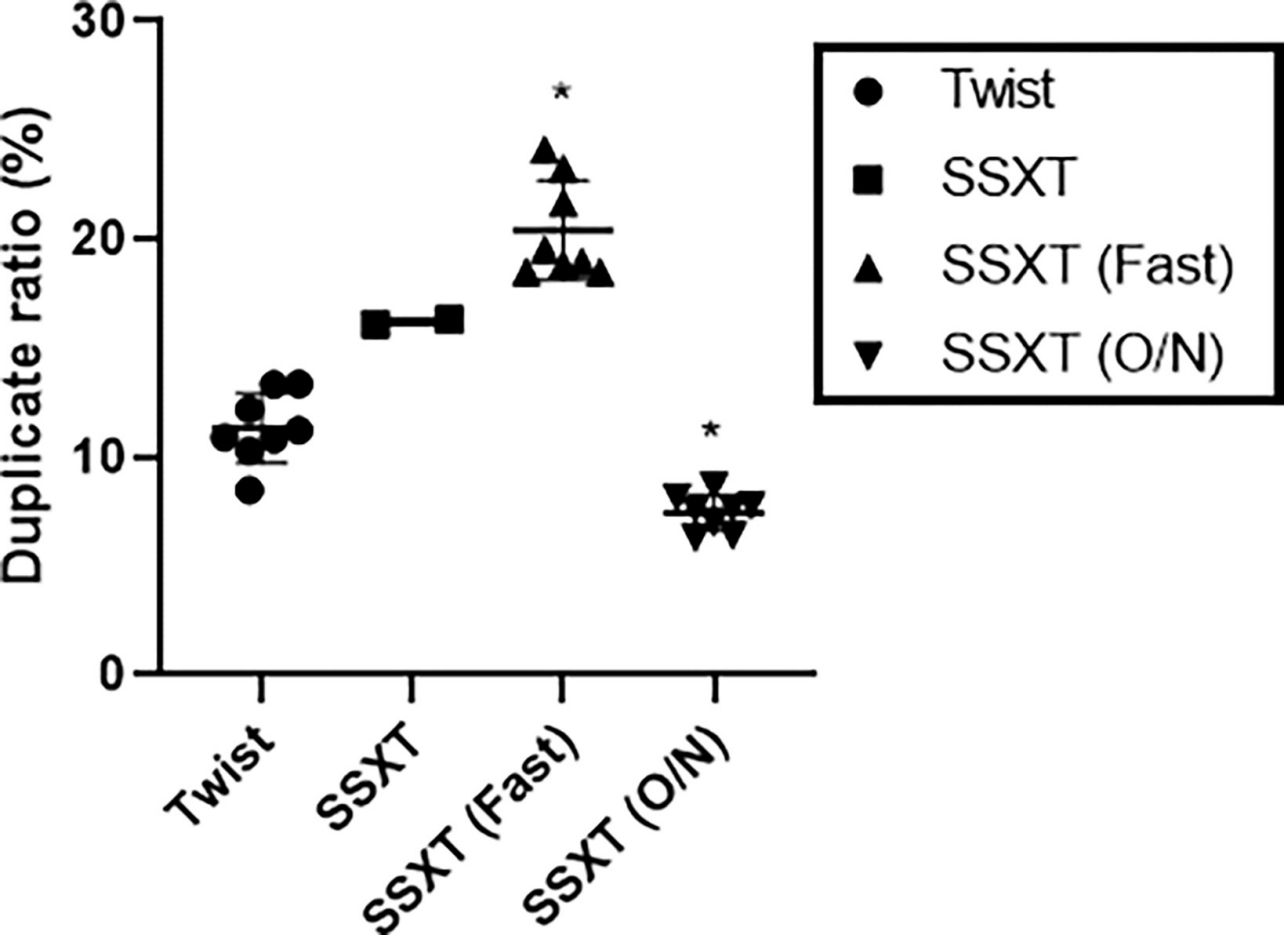
Duplicate ratios of the four whole-exome capture platforms. Duplicate ratio analysis was performed via Twist (n = 8), SSXT (n = 2), SSXT (Fast) (n = 8), and SSXT (O/N) (n = 8) kits. The means ± SDs were calculated from eight independent experiments (except for SSXT). *P < 0.05 compared with Twist.

Lower duplicate rates in PCR-based sequencing with probes typically result in higher data accuracy. Among the kits, the SSXT (Fast) kit had the highest duplication rate, while the SSXT (O/N) kit had the lowest level (Fig 3, S2 Table) (*P*<0.0001, *R*^*2*^: 0.9247). A comparison of the duplication of six buccal swabs and two blood samples from each breed revealed no obvious differences (S5 Table).

### Detection efficiency of the four whole-exome capture platforms for variant calling

**Fig 4:**
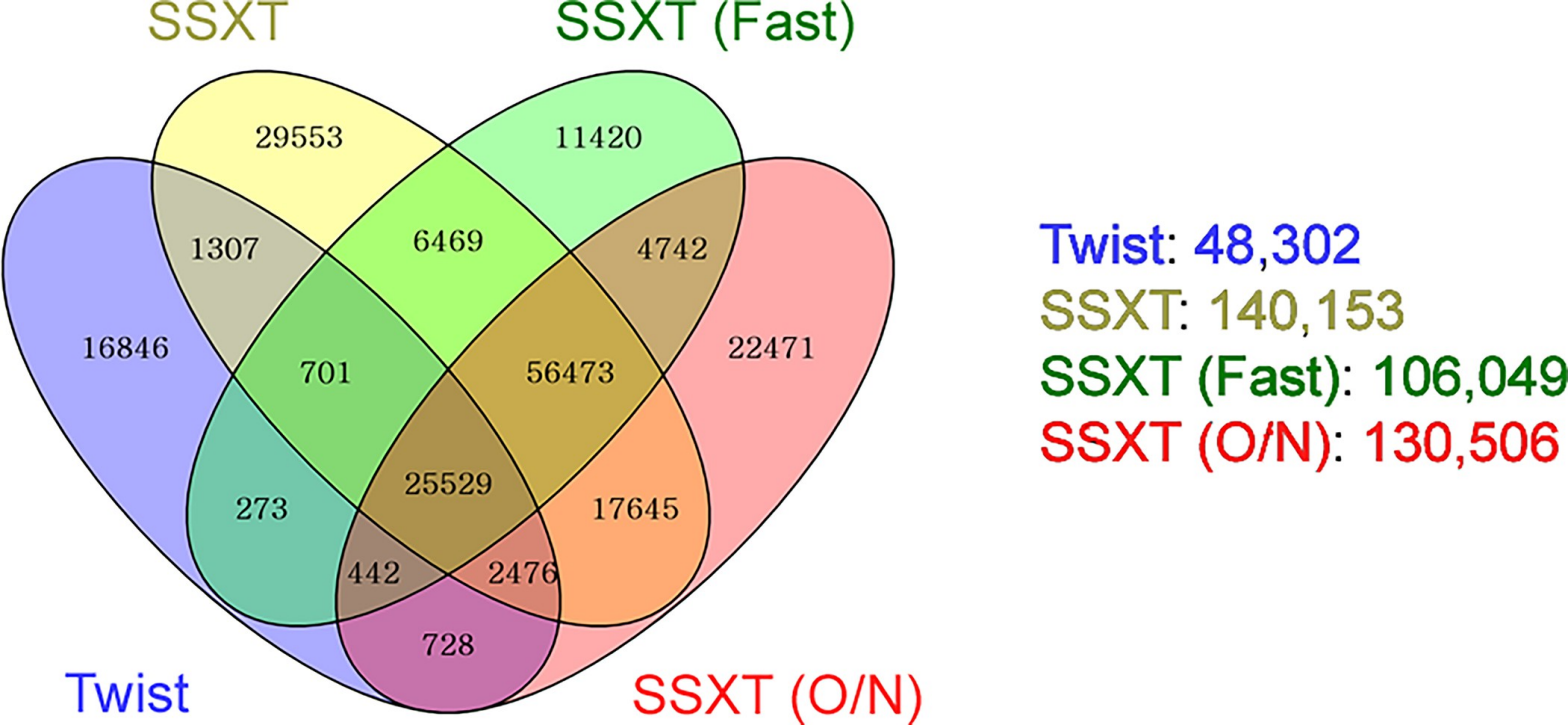
Detection efficiency of the four whole-exome capture platforms for variant calling. Variant detection analysis was performed via Twist (n = 8), SSXT (n = 2), SSXT (Fast) (n = 8), and SSXT (O/N) (n = 8) kits.

The primary objective of whole-exome sequencing (WES) is to detect as many variants as possible. Therefore, we compared the variant calling performance of the four platforms [12]. The SSXT kit had the highest number of variants detected at 140,153, followed by 106,049 in SSXT (Fast) and 130,506 in SSXT (O/N). In contrast, only 48,302 variants were identified in Twist (Fig 4, S4 Table). A comparison of the six buccal swabs and two blood samples from each breed revealed no significant differences (S5 Table).

## Discussion

As awareness about companion dogs grows, there is increasing interest in addressing their health issues [17]. While treating diseases with medication or surgery is important, prevention through disease prediction can be more effective, allowing companion dogs to lead a healthy life and spend quality time with their families. To meet these demands, the development of disease prediction methods through genome analysis is crucial [18]. WES is a powerful genome analysis method that can greatly increase the accuracy of results while reducing analysis costs [19]. Therefore, optimizing the most efficient WES analysis method is necessary to predict and prevent diseases effectively in companion dogs.

In our study, we conducted a comparative analysis of four whole-exome capture platforms from Agilent and Twist via the genome of a canine model. The comparison between Twist and Agilent kits revealed several key findings: 1) Comparison of experimental methods and characteristics of each kit revealed distinct differences; 2) the SSXT (O/N) kit demonstrated the best target coverage efficiency and accuracy levels; 3) the SSXT (O/N) kit presented an exceptionally low duplication rate compared with the other three kits; and 4) Agilent kits, especially the SSXT (O/N) and SSXT kits, detected more variants overall than did the Twist kit. These results emphasize the importance of optimizing the most effective WES analysis method for robust genome analysis in companion dogs.

Twist’s kit [https://www.twistbioscience.com/node/16736] stands out for its stability, even under long-term storage and repeated freezing and melting conditions during experiments, owing to its double-stranded DNA probe design. This kit utilizes a precapture probe method, allowing for the collection of up to eight DNA samples in a single tube and enabling a relatively simple library preparation process that can be completed in as little as half a day.

Agilent kits utilize single-stranded RNA probes, which have relatively lower stability than DNA probes do. However, these RNA probes can hybridize strongly with template DNA (reference library), enabling stable sequencing. Among Agilent WES kits [20], SSXT, the oldest developed, uses a postcapture probe method and is known for its excellent sequencing quality. However, it involves several experimental steps in library preparation and requires individual tubes for each sample, resulting in a relatively long experimental time of approximately one day. The SSXT (Fast) kit [https://www.agilent.com/en/product/nonhuman%20Genomics] is a product that addresses this issue by significantly reducing the experimental time, which is a disadvantage of SSXT. It employs a precapture probe method similar to the Twist kit, and the library preparation process has been dramatically shortened. Additionally, the probe binding time was reduced to 2.5 h, resulting in a shorter total experimental time. On the other hand, the SSXT (O/N) kit uses the same experimental method as SSXT (Fast) but allows for a longer hybridization time of ssRNA probes, ranging from 16–24 h, enabling even more stable sequencing.

In this study, the Agilent kits that utilized ssRNA probes presented higher total read scores than did the Twist kit that used dsDNA probes. Among the Agilent kits, SSXT (Fast) had the highest total read levels, with the exception of SSXT. Furthermore, when the read count was normalized to 1, the Twist kit showed a larger deviation, whereas the Agilent kits showed relatively smaller deviations. Among the Agilent kits, SSXT (O/N) demonstrated the best evenness, except for SSXT. The on-target ratios generally decreased as the sequencing depth increased, but the gentler the slope was, the greater the degree of sequencing coverage. The SSXT and SSXT (O/N) kits presented relatively high on-target ratios. However, the on-target ratio of the Twist kit initially ranked second highest after those of SSXT and SSXT (O/N) at low sequencing depths (2–20x) but rapidly decreased as the depth increased. These findings suggest that, overall, the SSXT (O/N) kit exhibited the best target coverage efficiency compared with Agilent SSXT as a reference.

To achieve high-accuracy sequencing results, it is important to minimize the generation rate of duplicate sequences. Among the kits compared, the SSXT (O/N) kit had the lowest rate of overlapping sequences (duplicates), resulting in more accurate sequencing data. Surprisingly, relatively poor levels of duplicate generations were observed with the SSXT and SSXT (Fast) kits, whereas the Twist kit presented the second lowest levels. These findings suggest that conducting experiments with sufficient hybridization time via the precapture ssRNA probe method may lead to relatively accurate sequencing results by reducing the generation rate of duplicates. The SSXT (O/N) kit was designed to meet these conditions.

The ultimate goal of commercial WES kits is to detect the maximum number of variants on the basis of high-quality sequencing data. In our evaluation, Agilent’s kits detected an average of 125,569 variants, with SSXT detecting the highest number of variants (140,153), followed by SSXT (O/N), with 130,506 variants. In contrast, Twist showed a relatively low detection ability, with only 48,302 variants.

In conclusion, our study demonstrated that the Agilent SSXT (O/N) kit provides accurate and efficient sequencing results for a canine exome, surpassing both the Twist and Agilent SSXT (Fast) kits in terms of variant detection, while also being relatively cost-effective.

## Supporting information

S1 Fig Schematic diagram of the complete process of the WES experiment and analysis. (TIF)

S1 Table Information on the experimental sets is organized. (XLSX)

S2 Table Comparison of SSXT and SSXT (O/N) kits. (XLSX)

S3 Table Comparison of Twist, SSXT (Fast), and SSXT (O/N) kits. (XLSX)

S4 Table Analysis of variants. (XLSX)

S5 Table A comparison of six buccal swabs and two blood samples from each breed. (XLSX)

## Abbreviations used

WES: Whole -exome sequencing
SSXT: SureSelect XT
SSXT (Fast): SureSelect XT HS2 (Fast hyb)
SSXT (O/N): SureSelect XT HS2 (Overnight hyb)
