# Supplementary figures and images for: Comparative analysis of whole exome sequencing kits for the canine genome
Source: PLoS One. 2024 Nov 4;19(11):e0312203. doi: 10.1371/journal.pone.0312203 (PMC11534239; doi:10.1371/journal.pone.0312203)

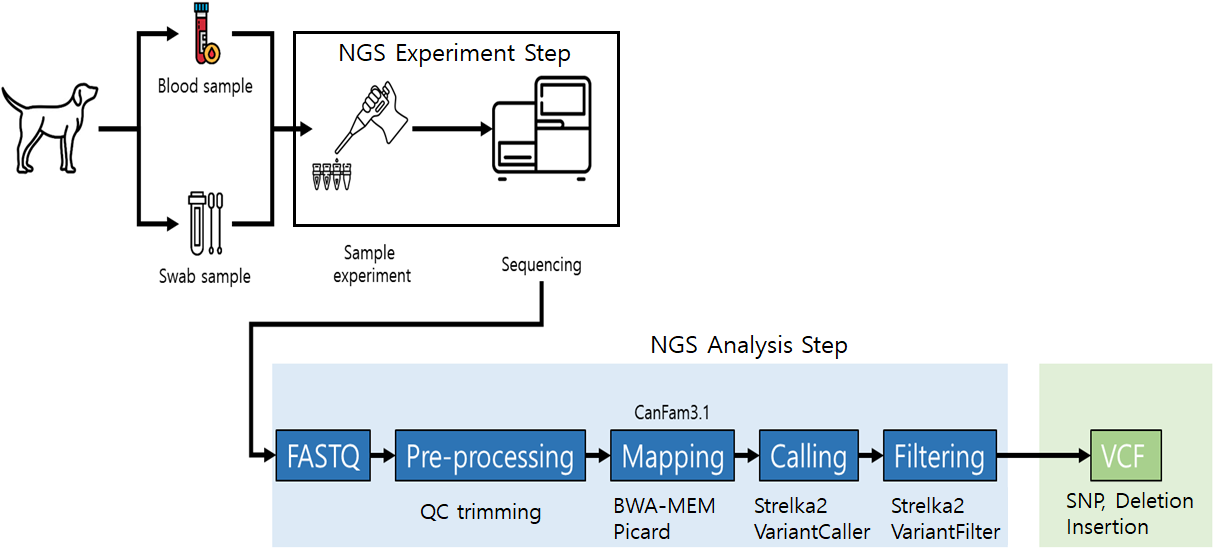

Supplement: S1 Fig — (TIF) [file pone.0312203.s001.tif]
